# A Case Report of Challenging Diagnosis of Persistent Hypoglycemia Secondary to Methadone Dose-Dependence in a Patient With End-Stage Renal Disease (ESRD) and Liver Cirrhosis (LC)

**DOI:** 10.7759/cureus.62903

**Published:** 2024-06-22

**Authors:** Nehemias Guevara, Volha Chapiolkina, Juan Pesantez, Loran Rakovica, Jamil Ibrahim

**Affiliations:** 1 Medicine, St. Barnabas Hospital Health System, New York, USA; 2 Critical Care Medicine, St. Barnabas Hospital Health System, New York, USA

**Keywords:** management, approach diagnosis, opioids use disorder, methadone, hypoglycemia

## Abstract

Methadone is a widely used opioid used for the management of chronic pain, treatment for opioid use disorders (heroin addiction), as well as for acute opioid withdrawal syndrome. Even though methadone is considered a safe drug, it is not exempt from unwanted side effects, some of them can be rare and may be overlooked due to patients’ comorbidities, delaying proper identification of the actual etiology. This article highlights one of those side effects, hypoglycemia, in a 64-year-old woman with a medical history of end-stage renal disease (ESRD) on hemodialysis, Acquired immune deficiency syndrome, liver cirrhosis, and a history of intravenous (IV) drug abuse disorder on a methadone maintenance program, and the importance of prompt identification and management, such as dose splitting or alternate medication to manage opioid withdrawal. The case underscores the importance of a proper approach and the high clinical suspicion that must be present when multiple variables are in place.

## Introduction

Opioids have been used for over 8,000 years [1]. We can classify opioids as natural and semi-synthetic compounds. In addition, opioids can be categorized depending on the receptor where they act to produce their effects and belong to the G-protein-coupled receptors; they are classified as mu, delta, and kappa. Opioid receptors are distributed broadly within the central nervous system and throughout the periphery, vas deferens, knee joint, gastrointestinal tract, heart, and immune system.

Nevertheless, opioids are not exempt from adverse events; the most concerning ongoing problem is the epidemic of opioid abuse that was declared in 2015 in the United States after 33,091 deaths due to overdose. Furthermore, the number of people using opioids for the first time has continued to grow [2]. In 2020, 91,799 drug overdose deaths occurred in the United States [3]. In 2020, more than six million opioid analgesic prescriptions were dispensed per the surveillance state system [4]. However, opioids are a cornerstone for managing different diseases, such as chronic non-cancerous pain, acute pain, and irritable bowel syndrome.

Hypoglycemia has been associated with opioids; some studies have shown it is receptor-related and that tramadol and methadone share the same probability of producing hypoglycemia [5,6]. Andrew J. Faskowitz et al. conducted experimental studies with mice, and methadone was dose-dependent inducing hypoglycemia [7]. Nevertheless, many questions still need to be answered regarding the mechanism, dose, and which receptors are specifically involved.

There are few cases reported regarding methadone-induced hypoglycemia. We are presenting here a case of a 64-year-old woman with end-stage renal disease (ESRD), and liver cirrhosis presenting with dose-dependent recurrent hypoglycemia in the setting of methadone use.

This article was previously presented as a meeting poster at the 2023 American College of Chest Physicians (CHEST) annual meeting on October 8-11, 2023.

## Case presentation

**Figure 1:**
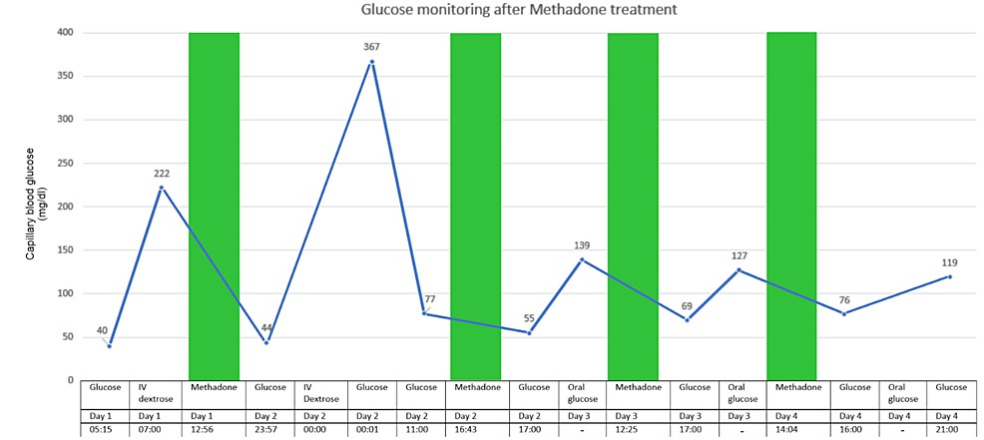
Glucose levels and methadone dose correlation Green bars: time at which methadone dose (140 mg oral) was given; Blue line: trend in glucose levels.

A 64-year-old woman with a medical history of ESRD on hemodialysis, Acquired immune deficiency syndrome, liver cirrhosis, and a history of IV drug abuse disorder on a methadone maintenance program presented to the emergency department (ED) with complaints of dizziness and weakness associated with an increase in sweating, and palpitations that started approximately one hour before arrival to the ED. Initial evaluation revealed blood glucose levels of < 26 mg/dl. The patient showed clinical improvement after receiving two boluses of 50 mg/dl IV dextrose to blood glucose of 65 mg/dl. During the hospital course, the patient had episodes of hypoglycemia and remained responsive to oral and intravenous (IV) glucose administration (Figure 1).

**Figure 2:**
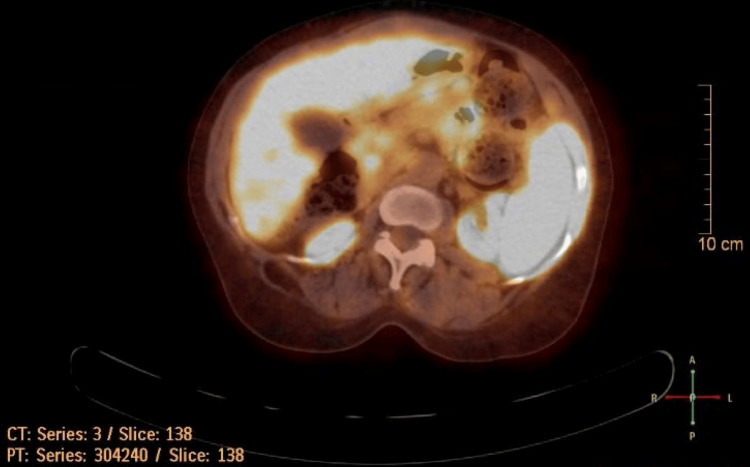
Positron emission tomography (PET) scan with a focus on the pancreas to rule out insulinoma

Imaging studies to evaluate for insulinoma including positron emission tomography (PET) (Figure 2) of the abdomen reported a small focus of increased tracer uptake at the head of the pancreas and somewhat heterogeneous tracer uptake seen at the liver with multiple small foci of increased tracer uptake that was inconclusive for insulinoma. Moreover, serum insulin levels were low during these episodes along with C-peptide levels, cortisol however was elevated (Table 1), Also, it is worth mentioning that the hypoglycemia episodes were unrelated to the hemodialysis sessions. These episodes occurred after methadone administration, independent of whether the patient received hemodialysis or not.

**Table 1:** Laboratory workup for secondary causes of hypoglycemia POCT: point-of-care testing

TEST	DATE	RESULT	REF. RANGE
Insulin	05/27/2021	1.3 uIU/mL	2.6 - 24.9 uIU/mL
Proinsulin	05/27/2021	0.4 pmol/L	0.0 - 10.0 pmol/L
C-peptide	05/27/2021	0.5 ng/mL	1.1- 4.4 ng/mL
POCT Glucose	05/27/2021	50 mg/dL	70 – 99 mg/dL
Cortisol	05/27/2021	29.2 ug/dL	05-23 5.0-23 ug/dL

**Figure 3:**
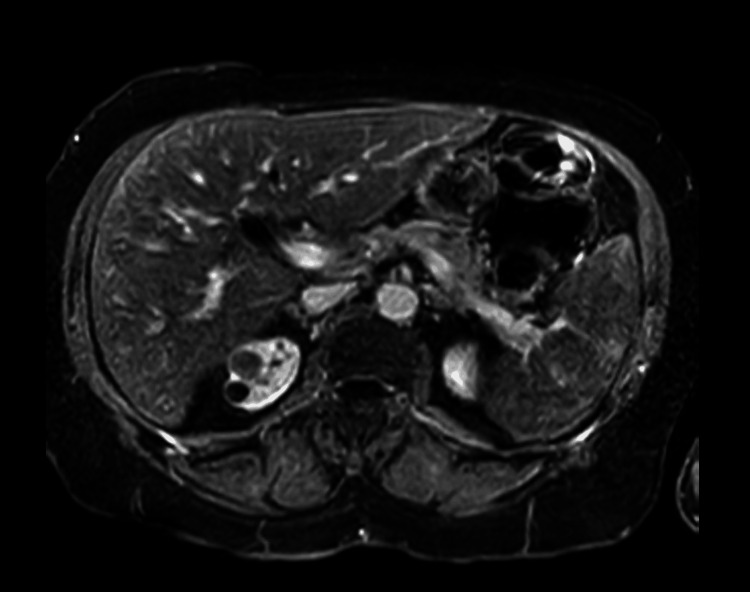
MRI of the abdomen for further visualization of the pancreas to rule out insulinoma

After discharge, the patient underwent a magnetic resonance imaging (MRI) of the abdomen with contrast as an outpatient. It showed an atrophic pancreas without any masses or dilated common bile duct (Figure 3).

During the current hospital course, the patient’s hypoglycemia was suspected due to a high dose of methadone, and a positive correlation was noticed when the patient was given the high methadone doses; furthermore, the Naranjo adverse drug reaction probability score was 10 [8]. 

The plan was made to switch from methadone to a different opioid-buprenorphine, but the patient refused to change her medication. The dose was split from 140 mg daily to 70 mg two times a day (BID), during which fewer hypoglycemic events were reported. After discharge, during the follow-up visits, the patient refused the split dose, and hence she was given oral diazoxide.

## Discussion

Methadone is a synthetic opioid known best for treating opioid dependence. Its favorable profile makes it a preferred agent for chronic pain management. However, potential adverse events and possible medication interactions in a population that is following a methadone maintenance treatment program for opioid dependence are poorly understood due to their underlying comorbidities [9]. Being a fat-soluble drug, methadone is rapidly absorbed orally, and its peak concentration reaches one to five hours, with an analgesic effect occurring 30-60 minutes after administration. Its effects are mainly mediated by μt receptors located centrally and peripherally and produce the common μ opioid effects: constipation, sedation, respiratory depression, and miosis. Moreover, it antagonizes the N-methyl-D-aspartate (NMDA) receptors, which contribute to treating neuropathic pain [10]. Methadone is a highly protein-bound drug, and particular medications may compete for binding sites, resulting in serum concentration alterations [11]. Furthermore, methadone has a high concentration in tissue which may be higher than in plasma levels - this, together plus the affinity to bind to proteins, may explain the high plasma half-life of the drug [12]. The metabolism of methadone largely relies on its metabolite inactivation and elimination by the liver, feces, and urine. For patients with renal impairment, feces elimination of methadone will increase, and no dose adjustment is necessary. Moreover, due to being highly protein-bound, hemodialysis does not affect clearance. Therefore, patients with impaired liver function may require additional methadone titration. A retrospective study by Flory et al. showed a clear association between methadone exposure and hypoglycemia in inpatients admitted to tertiary cancer centers for more than 48 hours, particularly with doses greater than 40 mg [13]. Prior evidence exists that opioids affect endocrine systems via the pancreatic effect or hypothalamic-pituitary-adrenal (HPA) axis [14]. Proposed mechanisms of hypoglycemia associated with opioid use are endogenous hyperinsulinemia, hypoadrenalism, reduced hepatic hypoglycemia, suppression of counter-regulatory hormones, glucagon, and epinephrine. Retrospective analysis reveals a significant association of hypoglycemia with tramadol and methadone in contrast to other opioids [5]. Secondary hypoadrenalism has been described with acute and chronic administration of methadone, tramadol, and fentanyl in animal studies of hypoglycemia induced by intrathecal morphine. Due to methadone’s long half-life, a mechanism related to the HPA axis and NMDA receptor antagonism causes serotonin and norepinephrine reuptake inhibition in the central nervous system [15]. Opioid effects in the central nervous system (CNS) may contribute to hypoglycemia-associated autonomic failure (HAAF) via opioid receptor activation in the thalamus and hypothalamus, areas responsible for glucose sensing [16].

Prior cases of methadone-induced hypoglycemia in populations with end-stage renal disease resolved with discontinuation of methadone, and subsequent follow-up provided evidence that methadone use in hypoglycemia initially presented as hyperinsulinism. Adequate tapering or transition to buprenorphine resolved the hypoglycemia. Retrospective studies showed that methadone lowered the average blood glucose levels by 5.7 mg/dL [13]. Nevertheless, even though our patients had another risk factor in developing hypoglycemia, such as ESRD, patients with ESRD developed hypoglycemia in the setting of poor oral intake, after hemodialysis, or because they are still using insulin as part of their diabetes treatment [17,18]. 

Therefore, this scenario was ruled out in our patient; furthermore, the Naranjo adverse drug reaction probability scale, was 10, meaning an indication of a strong positive correlation with methadone as the reason for hypoglycemia. As there is no protocol for treating methadone-induced hypoglycemia, in our case, halving the patient's dose in two separate doses significantly decreased the incidence of hypoglycemia. Switching the opioid agonist is also advisable as the alternatives would be less hypoglycemia-inducing.

## Conclusions

Our case highlights the lesser-known adverse effect of methadone, such as hypoglycemia, in a patient with multiple comorbidities that can eventually be behind his hypoglycemia events as well. Nevertheless, an extensive diagnostic workup, including imaging and laboratory tests, effectively ruled out insulinoma, ESRD, and cirrhosis as causative factors. Despite normal findings in these areas, the patient exhibited low serum insulin and C-peptide levels, prompting further investigation. Ultimately, a correlation between methadone dosage and hypoglycemia became evident, implicating methadone's pharmacokinetics, characterized by high tissue concentration and a prolonged half-life, in the hypoglycemic episodes. Moreover, recognizing hypoglycemia as a potential adverse effect of opioid therapy, especially methadone, is imperative for patient safety. Furthermore, there is no specific guideline on how to treat hypoglycemia associated with methadone. Therefore, implementing meticulous dose management and considering alternative opioids can help mitigate this risk. Our case hallmarked the critical need to create awareness in the medical community about this not-well-known adverse event of methadone, knowing the current ongoing opioid epidemic in the USA and continued research to understand and manage opioid-induced hypoglycemia effectively.

## References

[1] Bandyopadhyay S (2019). An 8,000-year history of use and abuse of opium and opioids: how that matters for a successful control of the epidemic? (P4.9-055). Neurology.

[2] Meyer R, Patel AM, Rattana SK, Quock TP, Mody SH (2014). Prescription opioid abuse: a literature review of the clinical and economic burden in the United States. Popul Health Manag.

[3] Hedegaard H, Miniño AM, Spencer MR, Warner M (2021). Drug overdose deaths in the United States, 1999-2020. NCHS Data Brief.

[4] (2023). New York State Opioid Annual Report 2021. https://www.health.ny.gov/statistics/opioid/data/pdf/nys_opioid_annual_report_2021.pdf.

[5] Makunts T, U A, Atayee RS, Abagyan R (2019). Retrospective analysis reveals significant association of hypoglycemia with tramadol and methadone in contrast to other opioids. Sci Rep.

[6] Chrétien B, Dolladille C, Hamel-Sénécal L (2019). Comparative study of hypoglycaemia induced by opioids. Is it a class effect?. Expert Opin Drug Saf.

[7] Faskowitz AJ, Kramskiy VN, Pasternak GW (2013). Methadone-induced hypoglycemia. Cell Mol Neurobiol.

[8] National Institute of Diabetes and Digestive and Kidney Diseases (2019). LiverTox: clinical and research information on drug-induced liver injury [Internet]. Roussel Uclaf Causality Assessment Method (RUCAM) in drug induced liver injury.

[9] Brown R, Kraus C, Fleming M, Reddy S (2004). Methadone: applied pharmacology and use as adjunctive treatment in chronic pain. Postgrad Med J.

[10] Inturrisi CE (2002). Clinical pharmacology of opioids for pain. Clin J Pain.

[11] Lotsch J, Skarke C, Tegeder I, Geisslinger G (2002). Drug interactions with patient-controlled analgesia. Clin Pharmacokinet.

[12] Ripamonti C, Bianchi M (2002). The use of methadone for cancer pain. Hematol Oncol Clin North Am.

[13] Flory JH, Wiesenthal AC, Thaler HT, Koranteng L, Moryl N (2016). Methadone use and the risk of hypoglycemia for inpatients with cancer pain. J Pain Symptom Manage.

[14] Aloisi AM, Buonocore M, Merlo L (2011). Chronic pain therapy and hypothalamic-pituitary-adrenal axis impairment. Psychoneuroendocrinology.

[15] Gorman AL, Elliott KJ, Inturrisi CE (1997). The d- and l-isomers of methadone bind to the non-competitive site on the N-methyl-D-aspartate (NMDA) receptor in rat forebrain and spinal cord. Neurosci Lett.

[16] Zhang C, Pfaff DW, Kow LM (1996). Functional analysis of opioid receptor subtypes in the ventromedial hypothalamic nucleus of the rat. Eur J Pharmacol.

[17] Gianchandani RY, Neupane S, Iyengar JJ, Heung M (2017). Pathophysiology and management of hypoglycemiain end-stage renal disease patients: a review. Endocr Pract.

[18] Yun JS, Park YM, Han K, Kim HW, Cha SA, Ahn YB, Ko SH (2021). Severe hypoglycemia and the risk of end stage renal disease in type 2 diabetes. Sci Rep.

